# The effect of oregano essential oil on the prevention and treatment of *Salmonella pullorum* and *Salmonella gallinarum* infections in commercial Yellow-chicken breeders

**DOI:** 10.3389/fvets.2022.1058844

**Published:** 2022-12-21

**Authors:** Ziheng Xu, Can Wang, Changcheng Li, Min Wang, Wenyan Chen, Chenyu Zhou, Ping Wei

**Affiliations:** ^1^Institute for Poultry Science and Health, Guangxi University, Nanning, Guangxi, China; ^2^School of Public Health and Management, Guang University of Chinese Medical, Nanning, Guangxi, China

**Keywords:** oregano essential oil, *S. pullorum*, *S. gallinarum*, body weight, antibiotic alternatives

## Abstract

In order to prevent pullorum disease and fowl typhoid in breeders, the use of oregano essential oil (OEO) was tested for the prevention and treatment of infections of multidrug-resistant *Salmonella pullorum* (SP) and *Salmonella gallinarum* (SG) in commercial Yellow-chicken breeders. In the challenge-protection experiment, commercial Hongguang-Black 1-day-old breeder chicks were randomly divided into four groups, including A (challenged, preventive dose), B (challenged, treatment dose), C (challenged, untreated), and D (unchallenged, untreated). Group A was supplemented with 200 μL/L OEO in the drinking water during the whole trial (1-35 days of age) and group B was supplemented with 400 μL/L OEO during 8–12 days of age, while groups C and D were kept as untreated controls. At 7 days of age, birds of groups A, B, and C were divided into two subgroups with equal number of birds (A_1_-A_2_, B_1_-B_2_, and C_1_-C_2_), and then subgroups A_1_, B_1_, and C_1_ were challenged with SP, while subgroups A_2_, B_2_, and C_2_ were challenged with SG. Clinical symptoms and death were observed and recorded daily. Every week during the experiment, serum antibodies against SP and SG of all the groups were detected by the plate agglutinate test (PAT). At the age of 35 days, all birds were weighed and necropsied, lesions were recorded and the challenging pathogens were isolated. The results showed that the positive rates of SP and SG isolation in groups A_1_, A_2_ and B_1_, B_2_ were significantly lower (*P* < 0.05) than those of groups C_1_ and C_2_, respectively, while groups A_1_ and A_2_ were slightly lower (*P* > 0.05) than those of groups B_1_ and B_2_. The average body weight (BW) of groups A_1_ and A_2_ were significantly higher (*P* < 0.05) than those of groups B_1_, B_2_ and C_1_, C_2_, respectively, but there was no significant difference (*P* > 0.05) with that of group D. The *r*-value between PAT positive and the recovery rates of *Salmonella* was 0.99, which means they are highly positively correlated. The results of this study demonstrated that the prevention dose (200μL/L) and the treatment dose (400 μL/L) of OEO supplemented in the drinking water could all effectively decrease infections of SP and SG and that the effect of the prevention was greater than that of the treatment and finally that the prevention could also significantly reduce the BW decline of birds challenged with SP and SG.

## Introduction

Pullorum disease (PD) and fowl typhoid (FT), respectively caused by *Salmonella pullorum* (SP) and *Salmonella gallinarum* (SG), are two major avian salmonellosis diseases that seriously harm the health of the breeders of the local, so called Yellow-chickens (1, 2). Yellow-chickens, the main breed of broiler production and supply in China, are becoming more popular with consumers because of the delicious flavor of the meat. This breed dominates other broilers in southern China by 5 billion birds annually and are produced in the free-range style (3, 4). PD and FT can reduce the hatching ability and bring serious economic losses to the poultry industry (5). Interestingly, after infection with SP and SG, some infected birds can recover from PD and FT and some adult birds may not show clinical disease symptoms. However, they are still the carriers of SP and SG and then become a repository of *Salmonella* to contaminate other healthy birds through horizontal and vertical transmission (6, 7). PD and FT have been mostly eradicated from commercial poultry flocks in the developed countries but they are still prevalent in the developing countries, especially in the Yellow-chickens (8), due to the style of husbandry, incomplete eradication and inadequate biosecurity (9, 10).

Traditionally, antibiotics have been used to control commercial poultry infection by SP and SG and the isolation of *Salmonella* with multidrug resistance (MDR) has been increasing dramatically in recent years. That presents a threat to both the poultry industry and to public health (11, 12). Reducing antibiotic usage is an inevitable trend, and added antibiotics have been banned in the feed in China since 2020 (13). Therefore, there has been increasing interest in the development of new, effective and nontoxic antimicrobial compounds as an alternative to traditional antibiotic use.

Currently, some studies found that plant essential oils are a potentially useful source of antimicrobial compounds. This is attributed to their availability, fewer side effects and their ability to synthesize aromatic substances, the majority of which are phenols or oxygen-substituted derivatives (14). Oregano essential oil (OEO), an extract of oregano, has been found to be amongst the most effective antimicrobial and antioxidant natural agents (15). OEO is composed of a variety of aromatic compounds including carvacrol, thymol, γ-terpinene, *p*-cymene, linalool, β-myrcene and so on (16). Especially, the carvacrol and thymol, the two main phenols of OEO, are principally responsible for the antimicrobial activity of the oil and there is no problem with drug-residues and drug-resistance while using this product (17). These aromatic compounds can penetrate the cell membranes of pathogenic bacteria, change the permeability of the mitochondrial membranes and prevent mitochondria from absorbing oxygen. So, the growth of bacteria is inhibited because the pathogenic cells suffocated and died (18). Research has found that OEO, as a feed additive, can replace antibiotics, maintain animal growth performance and improve disease resistance, which allows oregano to be potentially used extensively in animal husbandry (19). The dietary supplementation with OEO can inhibit or kill harmful intestinal pathogens and improve broiler chicken production performance (20). Mathlouthi found that OEO supplementation could inhibit the growth of *Escherichia coli, Salmonella indiana, Listeria innocua, Staphylococcus aureus* and *Bacillus subtilis* (21). In addition to its antimicrobial and antioxidant effects, OEO also has anticoccidial and antiparasitic efffects. These efffects were demonstrated by both the studies of M. Bozkurt and M. Mohiti-Asli et al. (22, 23).

However, no study to date has reported on the prevention and treatment of PD and FT, the most important salmonellosis in Yellow-chicken commercial breeders, by using OEO. Therefore, this study aims to evaluate the prevention and treatment effects of OEO against the challenge of SP and SG in commercial Yellow-chicken breeders.

## Materials and methods

### Ethical statement

The live animals described in this study were treated according to the National Guidelines to Humanitarian Governance of Laboratory Animals Welfare (National Development and Reform Commission of the People's Republic of China, 2006) and the animal experiment was approved by the Animal Welfare and the Animal Experimental Ethical Committee of Guangxi University (No. 2020-gxu-097). All the experimental procedures were conducted in accordance with the Animal Welfare Act and the Guide for the Care and Use of Laboratory Animals. The animals were sacrificed by carbon dioxide narcosis.

### Materials

The OEO product, composed of thymol and carvacrol in the concentrations of 5.1 and 0.12% (w/w), was kindly provided by Shanghai Huihai Huamao Industrial Co., Ltd., China. 1-day-old breeder chicks of Hongguang-Black, negative for SP and SG, from the grandparent flock, were kindly provided by Guangxi Hongguang Agricultural and Animal Husbandry Ltd., China. Plate agglutination test (PAT) antigens of SP and SG were purchased from Beijing Zhonghai Biotech Co. Ltd., China. *Salmonella* A to F group-specific diagnostic sera and *Salmonella* serotype-specific monovalent sera were purchased from the Ningbo Tianrun Bio-pharmaceutical Co. Ltd., China. The isolates of multidrug-resistant SP and SG (The MDR spectrum were 17 and 12 antimicrobials, respectively) used for the challenge were provided by the Institute for Poultry Science and Health, Guangxi University, China.

### Determination of the infection dose

Fifty-five 1-day-old parent-stock breeder chicks of Hongguang-Black chickens were divided into five groups (A–E) with 10 birds per group, plus a group F with five birds, which served as the unchallenged and untreated control. The birds have free access to feed and water during the whole experiment and the diet was the commercial feed without any antibiotics or additives. Before the experiment, we collected serum from each 1 day old chicks for the plate agglutination test using *Salmonella* A to F group-specific diagnostic sera and *Salmonella* serotype-specific monovalent sera. At the age of 7 days, birds in groups A to E were divided into two subgroups, equal in number, designated groups A_1_ to E_1_ and A_2_ to E_2_. Groups A_1_ to E_1_ were orally challenged with 2 × 10^6^ CFU/mL, 2 × 10^7^ CFU/mL, 2 × 10^8^ CFU/mL, 2 × 10^9^ CFU/mL and 2 × 10^10^ CFU/mL of SP in 0.5 mL-volume/bird respectively, while groups A_2_ to E_2_ were orally challenged with 2 × 10^6^ CFU/mL, 2 × 10^7^ CFU/mL, 2 × 10^8^ CFU/mL, 2 × 10^9^ CFU/mL and 2 × 10^10^ CFU/mL of SG in 0.5 mL-volume/bird respectively. The dose of challenge and detection index refers to the previous procedures of our laboratory (24). More than 80% infective dose of *Salmonella* infection in birds was determined, which referred to Guiding Principles for Clinical Efficacy Evaluation of Antimicrobial Drugs II and III (China Ministry of Agriculture Proclamation No. 1247) (25). It is pointed out in the guideline that the infection rate of 80% in the artificial infection model is considered to be successful in the construction of the model and more in line with the actual situation.

### The challenge-protection experiment

In this experiment, 105 1-day-old breeder chicks of Hongguang-Black chickens were randomly divided into groups A (challenged, preventive dose), B (challenged, treatment dose) and C (challenged, untreated), with 30 birds each, and D (unchallenged, untreated) with 15 birds (Supplementary Table 1). The birds of each group were housed separately in the isolators. The birds were raised according to the model established by the laboratory and throughout the entire experiment they had free access to feed and water (24).

During the entire trial of 35 days, all groups were fed the same feed as that used in the prior experiment of the infection dose. In the drinking water, 200 μL/L of OEO was added in group A during the whole trial, 400 μL/L of OEO was added in group B during the ages of 8–12 days, while no supplement was added in groups C and D. At the age of 7 days, birds in groups A, B, and C were divided into two subgroups respectively, equal in number, and designated as A_1_, A_2_, B_1_, B_2_, C_1_, and C_2_. Groups A_1_, B_1_, and C_1_ were orally challenged with 0.5 mL-volume/bird of the suspension containing 1 × 10^8^ CFU of SP, and groups A_2_, B_2_, and C_2_ were orally challenged with 0.5 mL-volume/bird of the suspension containing 1 × 10^9^ CFU of SG. The infective doses of SP and SG were determined by the prior experiment of the infective dose.

### Clinical symptoms, pathological changes and body weight (BW)

The clinical symptoms and the pathological changes of the possible dead birds during the experiment were observed and recorded daily. At the end of the experiment, at the age of 35 days, the live BW of all the birds in each group was determined and recorded.

### PAT of the serum antibodies against SP and SG

PAT was performed before challenge, and all the birds were negative. At 1, 2, 3, and 4 weeks post-challenge, the serum antibodies against SP and SG of the birds were detected by PAT with the antigens of SP and SG according to the described method and the positive rates of the groups were calculated (26).

### Recovery of the challenged bacteria

At the end of the experiment at the 35 days of age, all surviving birds were euthanized and their organs were excised aseptically. The gross lesions were observed and recorded. The cloaca swabs, cecum, and the mixture of visceral parenchyma organs including heart, liver and spleen were sampled, respectively, for the isolation of the challenge *Salmonella* according to the routine of China National Food Safety Standard Methods for Food Microbiological Examination-*Salmonella* (GB/T4789.4-2016).

### Statistical analysis

The Microsoft Excel function and the Statistical Program for Social Sciences (SPSS) 22.0 statistical software were used to process the test data. Factor analysis of variance and chi-square test were used to test the difference between groups. *P* < 0.05 indicated that the difference was significant, while *P* > 0.05 was not. Different letters indicate that the difference was significant in the **Figures 2**–**4** (*P* < 0.05), while the same letters were not. The correlation coefficient *r*-value was calculated by the CORREL formula used to evaluate the correlation between PAT positive and the recovery rate of *Salmonella*. The range of the *r*-value from −1 to 1 (*r* > 0 is a positive correlation, *r* < 0 is negative correlation, and *r* = 0 is irrelevant). The greater the absolute *r*-value is, the higher the correlation is.

## Results

### Optimal doses of the infection

According to the results of the experiment of the infection dose (Table 1), 80% of birds were infected by the challenge with 2 × 10^8^ CFU/mL of SP and 2 × 10^9^ CFU/mL of SG, and there was no bird death during the whole trial. The infective doses of SP and SG were 2 × 10^8^ CFU/mL and 2 × 10^9^ CFU/mL.

**Table 1:** The results of each group in the experiment of the infection dose.

** Challenged strains **	** Groups **	** Infective dose (CFU/mL) **	** Clinical symptom 1 **	**Salmonella isolation**	** PAT 2 **	** Infection rates 3 **
SP	A_1_	2 × 10^6^	20%	20%	40%	40%
	B_1_	2 × 10^7^	20%	20%	40%	60%
	C_1_	2 × 10^8^	40%	40%	60%	80%
	D_1_	2 × 10^9^	40%	80%	80%	100%
	E_1_	2 × 10^10^	60%	80%	100%	100%
SG	A_2_	2 × 10^6^	0%	20%	20%	20%
	B_2_	2 × 10^7^	0%	20%	40%	40%
	C_2_	2 × 10^8^	20%	20%	40%	40%
	D_2_	2 × 10^9^	40%	60%	80%	80%
	E_2_	2 × 10^10^	40%	80%	80%	100%

^1^Clinical symptom: white diarrhea. ^2^PAT, plate agglutinate test. ^3^Infection rates: infected/uninfected birds × 100%. Birds with either of clinical symptom, Salmonella isolation or PAT positive were infected.

### Clinical symptoms and anatomic lesions observed in the challenge-protection experiment

**Figure 1:**
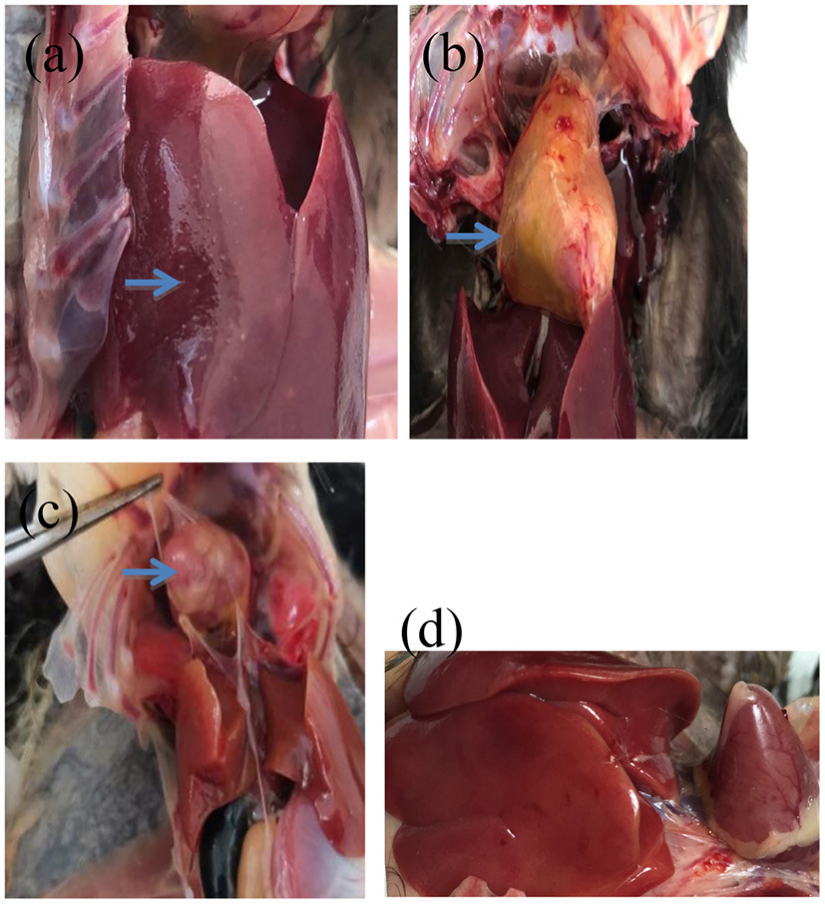
The anatomic lesions of the challenged birds. **(a)** Liver with necrotic white foci on the surface (arrow). **(b)** Heart with pericardial effusion containing yellow cellulose exudates (arrow). **(c)** Heart with granuloma (arrow). **(d)** Blank control.

According to clinical reports, the typical clinical symptoms of chicks were weakness, depressed appetite, poor growth, wet vent with chalky white droppings after *S. pullorum* and *S. gallinarum* infection. Birds in groups C_1_ and C_2_ showed weakness, ruffled feathers, loss of appetite, poor growth, and white diarrhea (Supplementary Figures 1A, B). The birds in A, B and C groups had the wet vent of chalky white material, and the number of birds in group C was the highest. At the necropsy of the birds, livers with necrotic white foci, as well as the softened heart with pericardial effusion containing yellow cellulose exudates, and granuloma of the heart could be observed in group C (Figures 1a–c); Livers with no lesions could be observed in group D (Figure 1d).

### Effect of OEO on the BW gain

**Figure 2:**
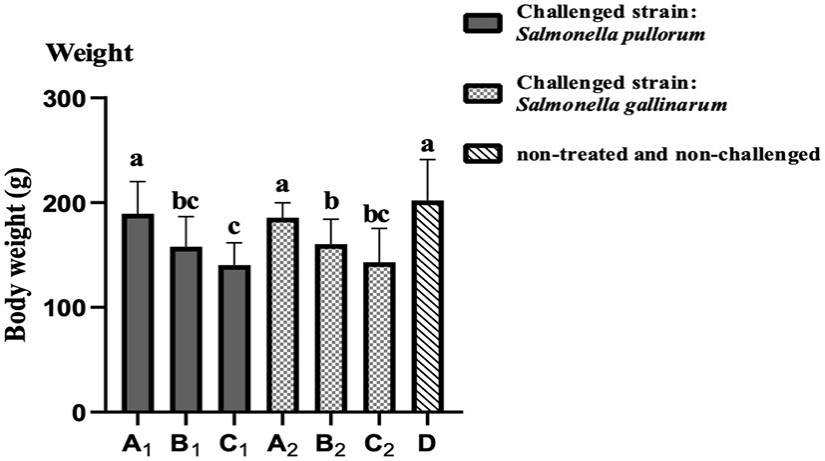
The average BW of the birds of different groups.

In the challenge-protection experiment, the average BW of birds in groups A_1_ and A_2_ were significantly greater (*P* < 0.05) than those of groups B_1_, B_2_ and C_1_, C_2_, respectively. Those of groups B_1_ and B_2_ were slightly greater (*P* > 0.05) than those of groups C_1_ and C_2_, respectively. And that of group D was significantly greater (*P* < 0.05) than those of groups B and C, but no significant difference (*P* > 0.05) was observed between groups D and A, even though that of group D was slightly greater than those of groups A_1_ and A_2_ (Figure 2).

### The result of PAT in the challenge-protection experiment

In the challenge-protection experiment, as shown in Table 2, the PAT positive rate of group A_1_ was significantly lower (*P* < 0.05) than that of group C_1_ at the end of the experiment (4 week post-challenge = 35 days of age), and no significant difference (*P* > 0.05) was found between groups A_1_ and B_1_, B_1_ and C_1_, even though that of group B_1_ was slightly higher than that of group A_1_ and was slightly lower than that of group C_1_. Also, the PAT positive rate of group A_1_ was found begun to be decreased from 3 weeks post-challenge and to become significantly decreased at 4 weeks post-challenge.

**Table 2:** PAT^1^ positive rate of each group in the challenge-protection experiment.

** Challenged strains **	** Groups **	** Weeks post-challenge (days of age) **			
		** 1 (14 d) **	** 2 (21 d) **	** 3 (28 d) **	** 4 (35 d) **
SP	A_1_	70%	70%^A2^	60%^A2^	40%^Aa2^
	B_1_	60%	60%	60%	50%^ab2^
	C_1_	90%	90%	90%	90%^b2^
SG	A_2_	70%^B2^	50%^B2^	50%	30%^Ba2^
	B_2_	70%	60%	50%	40%^a2^
	C_2_	100%	100%	100%	90%^b2^
–	Blank control	0.00%	0.00%	0.00%	0.00%

^1^PAT, plate agglutinate test. ^2^Means in the same column/row with different letters in the upper right are significantly different (P < 0.05) for each group.

Also, the PAT positive rates of groups A_2_ and B_2_ were significantly lower (*P* < 0.05) than that of group C_2_ at the end of the experiment, but no significant difference (*P* > 0.05) was observed between groups A_2_ and B_2_, even though that of group B_2_ was slightly higher than that of group A_2_. Also, the PAT positive rate of group A_2_ began to decrease from 2 weeks post-challenge and became significantly decreased at 4 weeks post-challenge. And the same trend was found in group B_2_ also.

### Recovery rate of the challenged SP and SG in the birds

**Figure 3:**
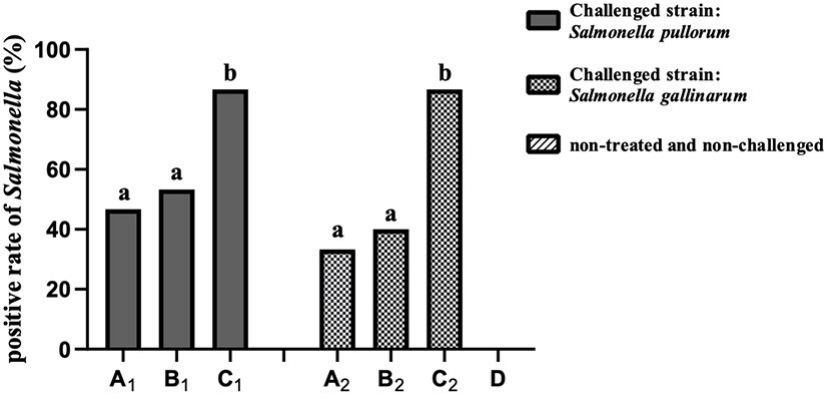
The total positive rates of *Salmonella* in the SP and SG challenge-protection experiments.

As shown in Figure 3, the total positive rates of *Salmonella* in groups A_1_ and B_1_ were significantly lower (*P* < 0.05) than that of group C_1_, and no significant difference (*P* > 0.05) was observed between groups A_1_ and B_1_. Similar results were found in the groups of the SG challenge-protection experiment. No *Salmonella* was isolated in group D.

**Figure 4:**
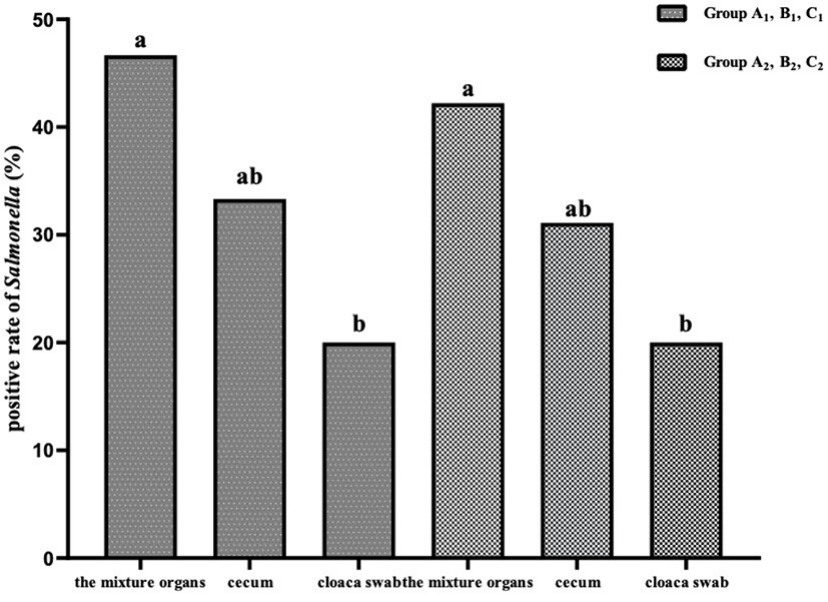
The positive rates of *Salmonella* in different organs in the challenge-protection experiment.

In both the SP and SG challenge-protection experiments, the positive rates of the mixture samples of the visceral parenchyma organs were significantly higher (*P* < 0.05) than that of cloaca swab, and slightly higher (*P* > 0.05) than that of the cecum (Figure 4).

### Comparison of the recovery rate of *Salmonella* and the PAT positive rate

As shown in Table 3, the total positive rate of PAT was higher than or equal to the recovery rate of the challenged *Salmonella*, and a group with a higher positive rate of PAT that also had a higher recovery rate of *Salmonella*. The *r*-value between these two tests was 0.99, which means they are highly positively correlated.

**Table 3:** Comparison of the positivity rate of the *Salmonella* and the PAT positivity.

** Challenged strains **	** Groups **	** Positive rate (%) **		
		**Salmonella isolation**	** PAT 1 **	** Both positive individual 2 **
SP	A_1_	46.67 (7/15)	46.67 (7/15)	5
	B_1_	53.33 (8/15)	53.33 (8/15)	6
	C_1_	86.67 (13/15)	86.67 (13/15)	12
SG	A_2_	33.33 (5/15)	40.00 (6/15)	3
	B_2_	40.00 (6/15)	46.67 (7/15)	5
	C_2_	86.67 (13/15)	86.67 (13/15)	11

^1^PAT, plate agglutinate test. ^2^Both positive individual: both the Salmonella isolation and the PAT were positive in the same bird.

## Discussion

FT and PD caused by SG and SP, respectively, are two serious septicemia diseases that greatly endanger the poultry industry and remain of major economic significance in many parts of the world (27). In this experiment, the birds manifested somnolescence, weakness, depressed appetite, poor growth, wet vent with chalky white droppings and livers with necrotic white foci, which are consistent with previous research (7). There was no bird death in the whole experiment, which was the same as in our previous study (24) and in the literature (28). Unsurprisingly, compared with other groups, no obvious clinical symptoms were observed in groups A_1_ and A_2_, indicating that there were better preventive effects of OEO against the challenges of SP and SG. To further understand the effects of OEO on SP and SG colonization in the challenged birds, the isolation and identification of the SP and SG in the internal organs and the intestine of birds were performed. The positive rates of isolation in groups C_1_ and C_2_ were significantly higher (*P* < 0.05) than those of groups A_1_ and A_2_ and B_1_ and B_2_, respectively, at the end of the experiment. The positive rates of SP and SG in groups A_1_ and A_2_ were slightly lower (*P* > 0.05) than those of groups of B_1_ and B_2_, respectively. The results showed that OEO could effectively prevent and treat the infection of SP and SG in chicks, and that the effect of prevention (supplemented during the whole experiment) was better than that of the treatment (supplemented during 1–5 days post-challenge). In addition, early studies have shown that the isolation rate in the liver was the highest, followed by the cecum and cloaca swabs (24, 29). In this study, the results showed that the positive rates of SP and SG in the mixture sample of the visceral parenchyma organs, which including heart, liver and spleen, was significantly higher (*P* < 0.05) than that of the cloaca swabs, and the positive rate of cecum was slightly higher (*P* > 0.05) than that of the cloaca swabs and was slightly lower (*P* > 0.05) than that of the mixture sample of the visceral parenchyma organs. These results were coincident with the previous studies (29). After the challenge with *Salmonella*, the liver, spleen and cecum are usually the main infected organs, accompanied by white foci of necrosis in the liver (30). The positive rate of cecum was lower than that of the mixture sample of the visceral parenchyma organs, which may be due to the intestinal antibacterial effect of OEO. Some research suggests that the barrier and antioxidant capacity of the intestine was improved by the oral administration of OEO, which may aide in gastrointestinal function without growth-promoting antibiotics (31). So the OEO used in this study may help to reduce the probability of *Salmonella* colonizing the intestine and may subsequently prevent the invasion of the pathogen into the internal organs.

In China, especially in Yellow-chickens, SP and SG were the most common serotypes of *Salmonella* infection (32). Therefore, it is important to control infection of SP and SG through implementation of an eradication program. The PAT is widely used on farms and on-site testing for eradication at the pre-maturation and the maturation stages of the breeders, being simpler and less costly than the isolation and identification of the bacteria (26, 29). In this study, at the end of the experiment (4 weeks post-challenge), the PAT positivity rates of groups A_1_ and A_2_ were lower than those of all other groups in the same challenged strains, and were significantly lower (*P* < 0.05) than those of groups C_1_ and C_2_, respectively. These results indicated that the preventive supplementation of OEO in the drinking water had a significant effect on the prevention of SP and SG in the birds. Meanwhile, the PAT positivity rates of groups A_1_ and B_1_ were slightly higher (*P* > 0.05) than those of groups A_2_ and B_2_, respectively, indicating that the effects of OEO on SG was better than those on SP. Interestingly, although the positivity numbers of serum PAT were the same or were more than those of the bacteria isolation in all the groups, there was still a high positive correlation between the results of these two tests. A few birds with the positive results of PAT might not be positive for the bacteria isolation and also a few birds with the negative results of PAT could be positive for the bacteria isolation. The two methods were not being completely equivalent, indicating that the PAT has certainly reference value for detecting *Salmonella* infection, but deviations may exist. Therefore, it is not possible to rely solely on the PAT for the detection of the positive birds. The positive birds should be confirmed by bacteriologic examination of 1 or more reactors (7). In routine testing, it is necessary to use the national standard method of bacteria isolation, for the detection of positive birds.

The published results highlight the bactericidal activity of OEO authorized in animal health as food supplements (33). OEO has the ability to improve the growth performance of poultry, increase the weight gain, improve the absorption capacity for nutrients, enhance the immune ability and reduce the infection of bacteria and diseases (19). In this study, at the end of the challenge-protection experiment (35 days of age), the BW of birds in group C was significantly lower (*P* < 0.05) than those in other groups, which may be due to the infection of *Salmonella* which can cause a severe inflammatory reaction in the intestinal mucosa of birds and can then lead to the decreased function of digestion and absorption and to the excessive requirement of nutrients in the process of resisting infection and then to poor growth performances (34). The BW of group A supplemented with 200 μL/L of OEO was significantly improved (*P* < 0.05) compared to that of group C and there was no significant difference (*P* > 0.05) compared with group D (the blank control). The BW of groups B_1_ and B_2_ were slightly greater (*P* > 0.05) than those of groups C_1_ and C_2_, respectively. It is inferred that the supplementation of OEO in the drinking water could help to improve the BW decline in birds due to *Salmonella* infection, and also revealed that the effect of the preventive supplementation was better than that of the treatment supplementation, even though the dose of the later was double that of the former. At the same time, compared with the effect of the treatment dose, the effect of the prevention dose was not only better in improving the BW decline caused by the infections of SP and SG, but also decreased the positive rates of the *Salmonella* isolation and the PAT. This might be related to the longer time of adding OEO in the groups of the preventive supplementation and the use of OEO before the bacteria challenges. Researchers have found that OEO can significantly promote the absorption of nutrients and regulate the balance of intestinal microflora (35, 36). Therefore, the preventive supplementation of OEO in the drinking water during the whole experiment could increase the BW gain performance of the challenged birds.

The OEO used in this study is mainly composed of thymol and carvacrol in the concentrations of 5.1 and 0.12% (w/w), respectively. Previous studies showed that the addition of a mixture of thymol and carvacrol can improve the production performance and antioxidant enzyme activity, delay the lipid oxidation, enhance the digestive enzyme activity and improve the immune response of broilers (37). Meanwhile, carvacrol and thymol, as the two main phenols of OEO, are principally responsible for the antimicrobial activity of the oil and there is no problem of a drug-residue issue in the products after using (16, 17). OEO can effectively kill bacteria without becoming resistant to them (38). Carvacrol and thymol can disturb the membrane integrity, increase membrane permeability and cause the leakage of protons and potassium, finally leading to the loss of membrane potential of the bacteria (39). So, the growth of bacteria is inhibited because of the depletion of the ATP pool and collapse of the proton motive force subsequently leading to the pathogen cells death (40). Therefore, this experiment proved that carvacrol and thymol might play an important role in the prevention of *Salmonella* (SP and SG) with OEO.

## Conclusion

In summary, the supplementation of OEO in the drinking water could effectively reduce the infection of *Salmonella* and its effect on SG was better than that on SP. Further, OEO could significantly improve the BW decline in birds infected by SP and SG. Also, the effect of the preventive supplementation was better than that of the treatment supplementation in the commercial Yellow-chickens.

## Data availability statement

The original contributions presented in the study are included in the article/Supplementary material, further inquiries can be directed to the corresponding author.

## Ethics statement

The animal study was reviewed and approved by the Animal Welfare and the Animal Experimental Ethical Committee of Guangxi University (protocol code 2020-gxu-097).

## Author contributions

Conceptualization and methodology: ZX and CW; Software, formal analysis, and investigation: CW and ZX; Resources and data curation: CW, CZ, and WC; Writing–original draft preparation: ZX, CW, CZ, and CL; Writing–review & editing and visualization, supervision, project administration, and funding acquisition: PW. All authors have read and agreed to the published version of the manuscript.
