# Supplementary material for: The effect of oregano essential oil on the prevention and treatment of Salmonella pullorum and Salmonella gallinarum infections in commercial Yellow-chicken breeders
Source: Front Vet Sci. 2022 Dec 21;9:1058844. doi: 10.3389/fvets.2022.1058844 (PMC9812558; doi:10.3389/fvets.2022.1058844)
Supplement: Supplementary file 1 [file Table_1.DOCX]

**Table S1. Arrangement of the challenge-protection experiment.**

| Groups | | Supplement in the drinking water | Challenged strains | No. Birds |
| --- | --- | --- | --- | --- |
| A | A1 | 200μL/L of OEO added  during the whole trial | SP | 15 |
|  | A2 |  | SG | 15 |
| B | B1 | 400μL/L of OEO added during the age  of 8-12 days | SP | 15 |
|  | B2 |  | SG | 15 |
| C | C1 | No OEO in the drinking water | SP | 15 |
|  | C2 |  | SG | 15 |
| D | Blank control | No OEO in the drinking water | — | 15 |

**Table S2. The results of each group in the experiment of the infection dose.**

| Challenged strains | Groups | Infective dose  (CFU/mL) | Clinical symptom^1^ | *Salmonella* isolation | PAT^2^ | Infection rates^3^ |
| --- | --- | --- | --- | --- | --- | --- |
| SP | A_1_ | 2×10^6^ | 20% | 20% | 40% | 40% |
|  | B_1_ | 2×10^7^ | 20% | 20% | 40% | 60% |
|  | C_1_ | 2×10^8^ | 40% | 40% | 60% | 80% |
|  | D_1_ | 2×10^9^ | 40% | 80% | 80% | 100% |
|  | E_1_ | 2×10^10^ | 60% | 80% | 100% | 100% |
| SG | A_2_ | 2×10^6^ | 0% | 20% | 20% | 20% |
|  | B_2_ | 2×10^7^ | 0% | 20% | 40% | 40% |
|  | C_2_ | 2×10^8^ | 20% | 20% | 40% | 40% |
|  | D_2_ | 2×10^9^ | 40% | 60% | 80% | 80% |
|  | E_2_ | 2×10^10^ | 40% | 80% | 80% | 100% |

^1^ Clinical symptom: white diarrhea.

^2^ PAT: plate agglutinate test.

^3^ Infection rates: infected/uninfected birds ×100%. Birds with either of clinical symptom, *Salmonella* isolation or PAT positive were infected.

**Table S3. PAT^1^ positive rate of each group in the challenge-protection experiment.**

| Challenged strains | Groups | Weeks post-challenge (days of age) | | | |
| --- | --- | --- | --- | --- | --- |
|  |  | 1 (14 d) | 2 (21 d) | 3 (28 d) | 4 (35 d) |
| SP | A_1_ | 70% | 70%^A2^ | 60%^A2^ | 40%^Aa2^ |
|  | B_1_ | 60% | 60% | 60% | 50%^ab2^ |
|  | C_1_ | 90% | 90% | 90% | 90%^b2^ |
| SG | A_2_ | 70%^B2^ | 50%^B2^ | 50% | 30%^Ba2^ |
|  | B_2_ | 70% | 60% | 50% | 40%^a2^ |
|  | C_2_ | 100% | 100% | 100% | 90%^b2^ |
| - | Blank control | 0.00% | 0.00% | 0.00% | 0.00% |

^1^ PAT: plate agglutinate test.

^2^ Means in the same column / row with different letters in the upper right are significantly different (*P*<0.05) for each group.

**Table S4. Comparison of the positivity rate of the *Salmonella* and the PAT positivity.**

| Challenged  strains | Groups | Positive rate (%) | | |
| --- | --- | --- | --- | --- |
|  |  | *Salmonella* isolation | PAT^1^ | Both positive individual^2^ |
| SP | A_1_ | 46.67 (7/15) | 46.67 (7/15) | 5 |
|  | B_1_ | 53.33 (8/15) | 53.33 (8/15) | 6 |
|  | C_1_ | 86.67 (13/15) | 86.67 (13/15) | 12 |
| SG | A_2_ | 33.33 (5/15) | 40.00 (6/15) | 3 |
|  | B_2_ | 40.00 (6/15) | 46.67 (7/15) | 5 |
|  | C_2_ | 86.67 (13/15) | 86.67 (13/15) | 11 |

^1^ PAT: plate agglutinate test.

^2^ Both positive individual: both the *Salmonella* isolation and the PAT were positive in the same bird.
