# Supplementary material for: The effect of oregano essential oil on the prevention and treatment of Salmonella pullorum and Salmonella gallinarum infections in commercial Yellow-chicken breeders
Source: Front Vet Sci. 2022 Dec 21;9:1058844. doi: 10.3389/fvets.2022.1058844 (PMC9812558; doi:10.3389/fvets.2022.1058844)
Supplement: Supplementary file 2 [file Data_Sheet_1.DOC]

.
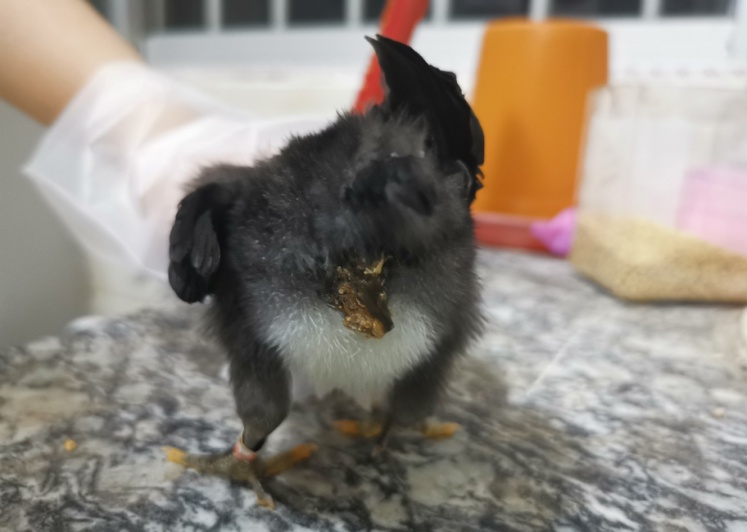

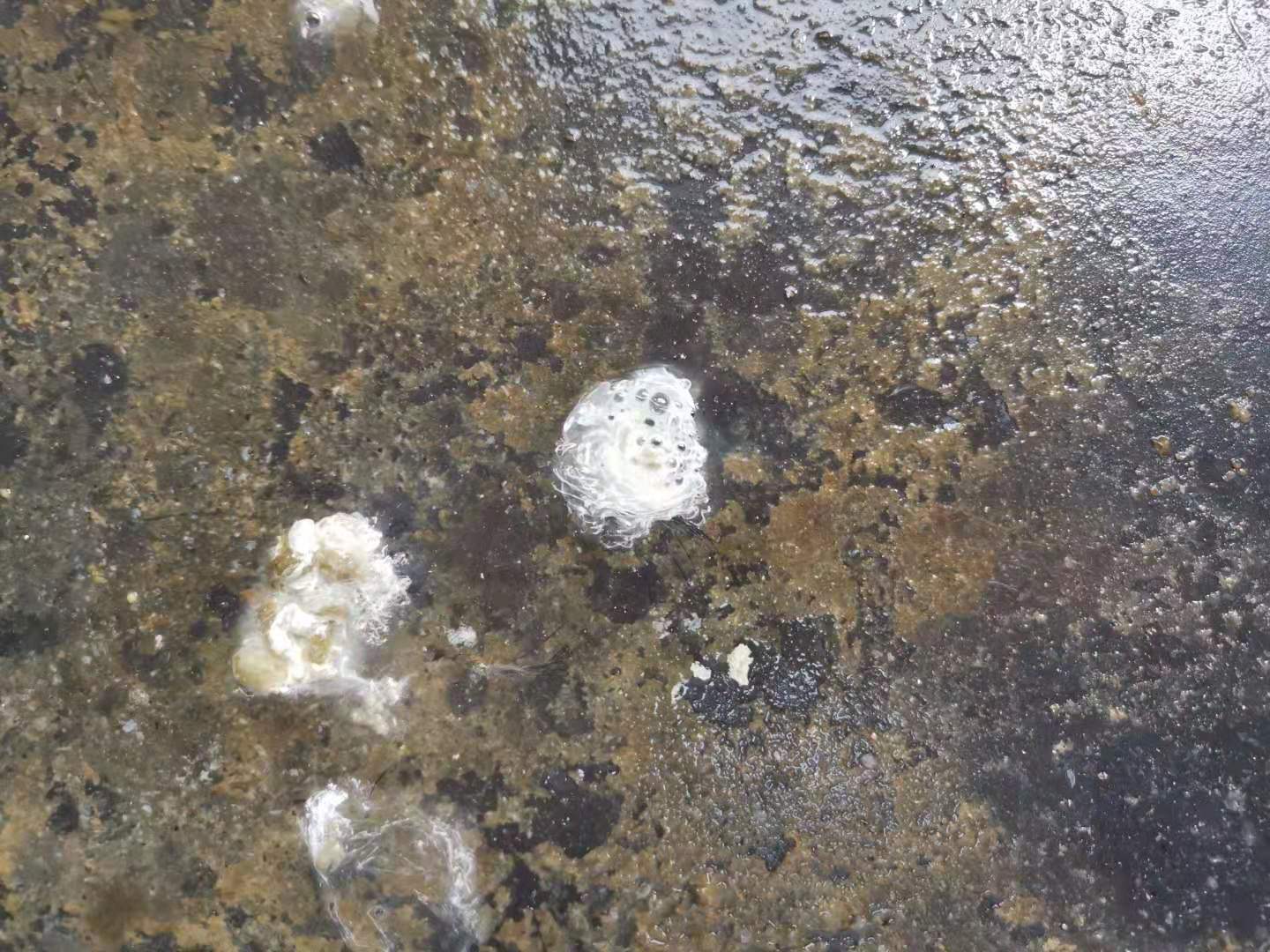


(a)

(b)

**Figure S1. The clinical observations of the challenged birds.**

(a) Wet vent of chalky white material. (b) White droppings.


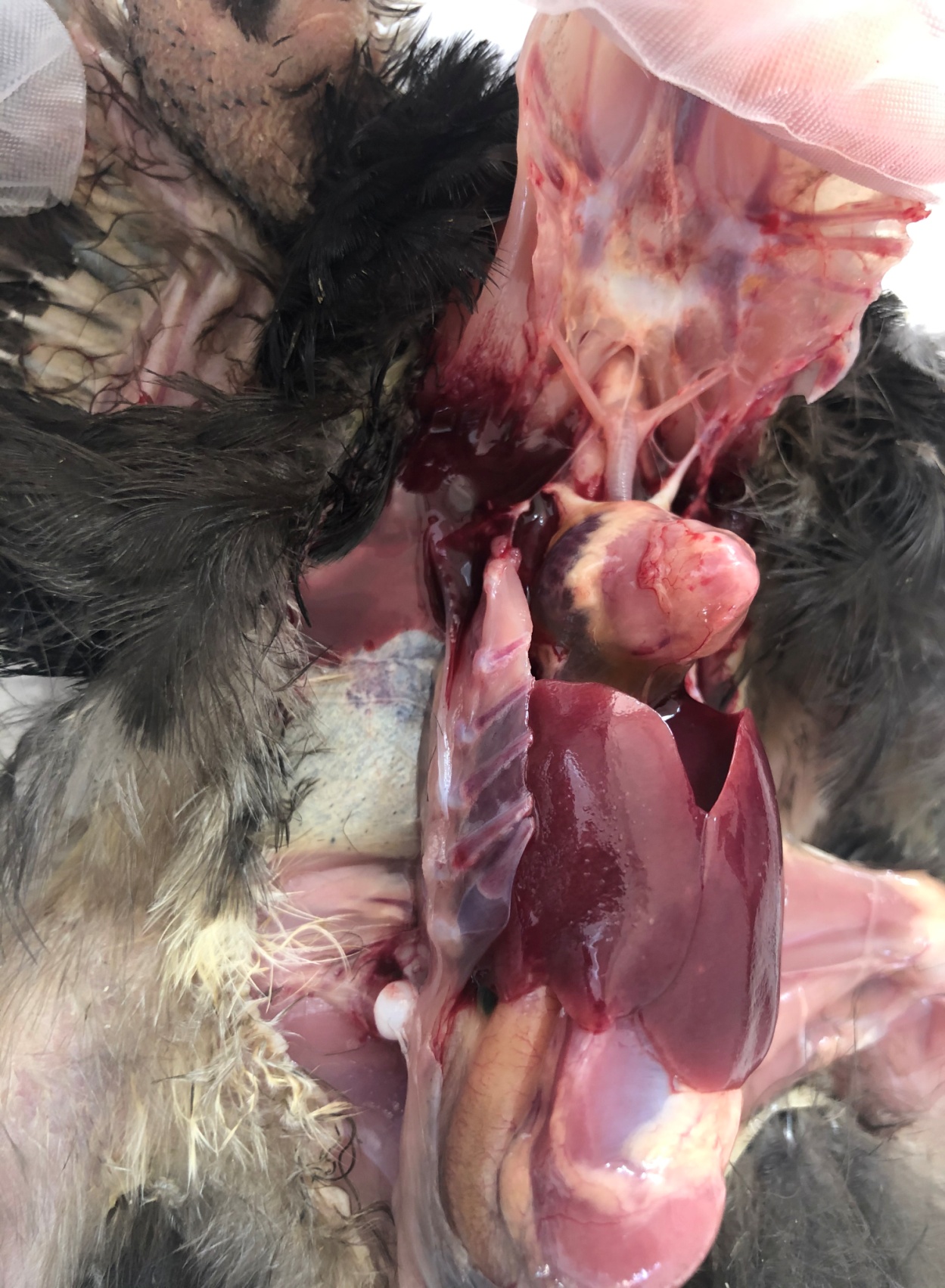

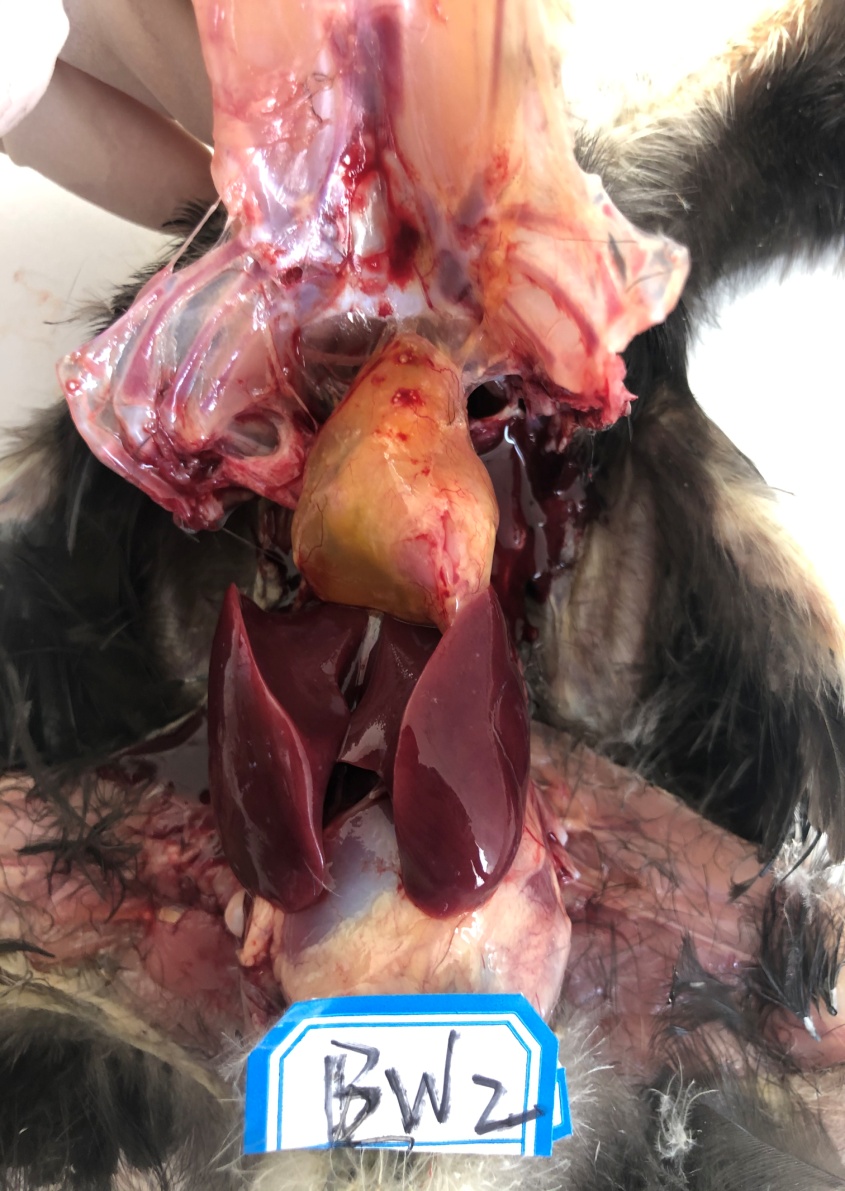

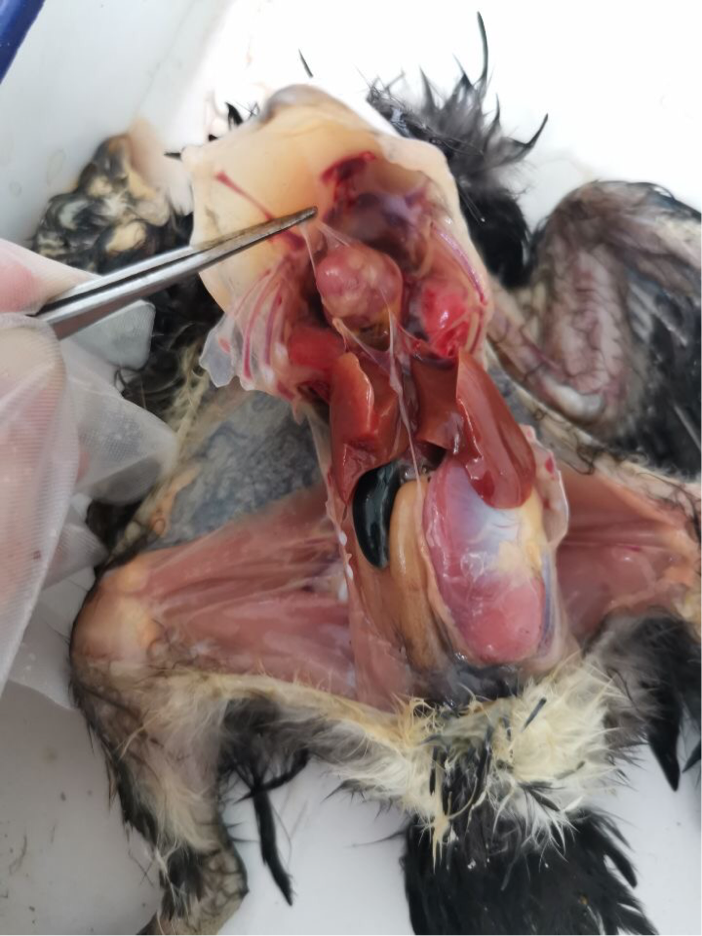


(b)

(c)

(a)

**Figure S2. The anatomic lesions of the challenged birds.**

(a) Liver with necrotic white foci on the surface (arrow).

(b) Heart with pericardial effusion containing yellow cellulose exudates (arrow).

(c) Heart with [granuloma](https://fanyi.so.com/?src=onebox#granuloma) (arrow)*.*
